# Network Pharmacology and Single‐Cell Transcriptomic Investigation of Molecular Mechanisms Underlying *Erigeron breviscapus* Treatment in Acute Myocardial Infarction

**DOI:** 10.1155/ijog/7715750

**Published:** 2026-05-31

**Authors:** Lili Yan, Jun Cao, Qinglong Xie, Lei Chen, Yaqin Shi

**Affiliations:** ^1^ Department of Cardiovascular Medicine, Taizhou Central Hospital, No. 999 Donghai Avenue Jiaojiang, Taizhou, 318000, Zhejiang, China, tzzxyy.com; ^2^ Department of Cardiovascular Medicine, Hubei Provincial Hospital of Integrated Chinese and Western Medicine, No. 11 Lingjiao Lake Road Jianghan, Wuhan, 430000, Hubei, China; ^3^ Department of Traditional Chinese Medicine, The Second People’s Hospital of Nantong, No.298 Xinhua Road Chongchuan, Nantong, 226002, Jiangsu, China, ahnmc.com

**Keywords:** acute myocardial infarction, *Erigeron breviscapus*, network pharmacology, single-cell transcriptomics, traditional Chinese medicine

## Abstract

**Background:**

Acute myocardial infarction (AMI) represents a life‐threatening cardiovascular emergency characterized by myocardial necrosis resulting from prolonged ischemia. *Erigeron breviscapus* (Vant.) Hand.‐Mazz. (EB), a traditional Chinese medicinal herb, has demonstrated cardioprotective properties in clinical applications. This investigation aims to establish a predictive computational framework for investigating the molecular mechanisms through which EB exerts therapeutic effects in AMI employing integrated network pharmacology and single‐cell transcriptomic strategies.

**Methods:**

Active pharmaceutical constituents of EB and their corresponding molecular targets were systematically retrieved from TCMSP and SwissTargetPrediction databases. AMI‐associated genes were compiled from GeneCards, OMIM, and DisGeNET repositories. Protein–protein interaction (PPI) networks were constructed utilizing STRING database, with core targets identified through topological analysis. Gene Ontology (GO) and Kyoto Encyclopedia of Genes and Genomes (KEGG) pathway enrichment analyses were performed to characterize biological functions. Single‐cell RNA sequencing (scRNA‐seq) data from myocardial infarction specimens were processed using 10× Genomics platform. Comprehensive analytical workflows included Seurat‐based clustering algorithms, UMAP dimensionality reduction, differential expression profiling, functional enrichment characterization, and Monocle 3‐mediated pseudotime trajectory reconstruction. Experimental validations encompassed murine AMI models, cellular senescence assays, and molecular interaction studies.

**Results:**

Network pharmacology analysis identified 45 bioactive compounds from EB corresponding to 286 potential therapeutic targets. Cross‐referencing with AMI‐related genes yielded 127 overlapping targets, suggesting multitarget pharmacological interventions. PPI network topology analysis revealed hub genes including VEGFA, AKT1, TNF, IL6, and PTGS2, indicating predicted involvement in angiogenesis, inflammation, and apoptotic pathways. KEGG enrichment demonstrated significant associations with PI3K‐AKT, MAPK, and TNF signaling cascades. Single‐cell transcriptomic profiling successfully delineated heterogeneous cardiac cell populations comprising cardiomyocytes, cardiac fibroblasts, endothelial cells, macrophages, and T lymphocytes. Differential expression analysis revealed substantial transcriptional reprogramming in post‐infarction myocardium, particularly within macrophage and fibroblast populations. Pseudotime trajectory analysis elucidated dynamic cellular state transitions during myocardial repair processes. Integration of network pharmacology predictions with scRNA‐seq data demonstrated that predicted EB target genes exhibited preferential expression in inflammation‐associated cell types. Functional enrichment of differentially expressed genes highlighted extracellular matrix reorganization, inflammatory response modulation, and neovascularization as principal biological processes potentially targeted by EB intervention.

**Conclusion:**

Through systematic integration of network pharmacology and single‐cell transcriptomics, this investigation established a predictive computational framework characterizing the multicomponent, multitarget, multipathway mechanisms underlying EB‐mediated cardioprotection in AMI. The findings provide computational evidence and hypothesis‐generating insights supporting EB therapeutic applications and establish a foundation for future experimental validation and precision medicine approaches in AMI management.

## 1. Introduction

Acute myocardial infarction (AMI) constitutes a predominant cause of cardiovascular mortality worldwide, characterized by irreversible myocardial injury secondary to sustained coronary occlusion and tissue hypoxia [1]. Global epidemiological surveillance indicates escalating AMI incidence rates, rising from 27.3% to 31.4% over recent decades, presenting substantial public health challenges [2]. The pathophysiological cascade of AMI encompasses multifaceted processes including cardiomyocyte death, inflammatory infiltration, extracellular matrix remodeling, and compensatory fibrosis, collectively contributing to adverse cardiac remodeling and progressive heart failure [3]. Contemporary therapeutic interventions, including pharmacological thrombolysis, percutaneous coronary intervention (PCI), and coronary artery bypass grafting (CABG), primarily focus on restoring coronary perfusion, yet demonstrate limited efficacy in preventing long‐term myocardial deterioration [4]. Consequently, elucidating novel therapeutic strategies targeting the molecular pathogenesis of post‐infarction injury represents a critical unmet medical need.

Traditional Chinese medicine (TCM) has garnered increasing recognition for cardiovascular disease management, offering potential advantages through multicomponent synergistic actions and holistic regulatory effects. *Erigeron breviscapus* (Vant.) Hand.‐Mazz. (EB), commonly designated as Dengzhanhua in Chinese pharmacopoeia, belongs to the Compositae family and has been extensively utilized for treating cerebrovascular and cardiovascular disorders in clinical practice. Phytochemical investigations have identified diverse bioactive constituents in EB, including flavonoids (particularly scutellarin and apigenin), phenolic acids, and terpenoids, which collectively contribute to its pharmacological activities [5]. Accumulating experimental evidence demonstrates that EB exhibits multifaceted cardioprotective properties encompassing antioxidative, anti‐inflammatory, antithrombotic, and proangiogenic effects. Specifically, scutellarin, the principal active component, has been shown to ameliorate myocardial ischemia‐reperfusion injury through modulating oxidative stress pathways and preserving mitochondrial function [6]. Clinical studies have documented EB’s efficacy in improving cardiac function indices and reducing adverse cardiovascular events in AMI patients [7]. However, the comprehensive molecular mechanisms underlying EB’s therapeutic actions in AMI remain incompletely characterized, necessitating systematic investigation.

Network pharmacology has emerged as a powerful systems biology approach for deciphering the complex mechanisms of TCM interventions, enabling systematic identification of drug–target–disease interactions through integrated computational analyses. This methodology aligns conceptually with TCM’s holistic philosophy of multicomponent, multitarget therapeutic paradigms. Recent advances in single‐cell transcriptomics have revolutionized our understanding of cellular heterogeneity and dynamic transcriptional landscapes in disease pathogenesis, providing unprecedented resolution for dissecting cell type–specific molecular alterations [8]. The integration of network pharmacology predictions with single‐cell transcriptomic validation represents an innovative strategy for comprehensively elucidating TCM mechanisms of action at cellular and molecular levels [9].

In the present investigation, we employed an integrated research framework combining network pharmacology analysis and single‐cell RNA sequencing (scRNA‐seq) to systematically explore the potential molecular mechanisms of EB in AMI treatment. Through comprehensive bioinformatics workflows, we identified EB’s bioactive constituents, predicted therapeutic targets, constructed molecular interaction networks, and performed functional enrichment analyses. Subsequently, single‐cell transcriptomic profiling of myocardial infarction (MI) specimens enabled characterization of cardiac cellular heterogeneity, identification of cell type–specific transcriptional alterations, and mapping of network pharmacology predictions to specific cell populations. It should be noted that the scRNA‐seq dataset employed in this study was derived from untreated MI samples without EB intervention, and thus the single‐cell analysis serves to identify potential cellular contexts for EB action rather than directly demonstrating drug‐induced changes. This multidimensional computational approach aims to establish a hypothesis‐generating framework for investigating EB’s cardioprotective mechanisms and establish a foundation for future experimental validation and clinical optimization in AMI management.

## 2. Materials and Methods

### 2.1. Network Pharmacology Analysis

#### 2.1.1. Active Compound Screening and Target Prediction

Bioactive constituents of EB were systematically retrieved from the Traditional Chinese Medicine Systems Pharmacology (TCMSP) database (http://tcmspw.com/tcmsp.php) employing stringent pharmacokinetic criteria: oral bioavailability (OB) ≥ 30% and drug‐likeness (DL) ≥ 0.18, ensuring favorable pharmaceutical properties. These threshold criteria were selected based on established TCMSP database conventions for identifying compounds with drug‐like properties and favorable pharmacokinetic characteristics, though we acknowledge these relatively lenient criteria may introduce false positives that require subsequent experimental validation. Molecular targets corresponding to selected active compounds were predicted using SwissTargetPrediction platform (http://www.swisstargetprediction.ch/) with probability scores > 0.5 indicating reliable predictions. This moderate prediction threshold balances sensitivity and specificity in target identification, while recognizing that computational predictions require experimental confirmation. Predicted targets were standardized to official gene symbols through UniProt database annotation.

#### 2.1.2. AMI‐Related Gene Collection

AMI‐associated disease genes were comprehensively compiled from three authoritative databases: GeneCards (https://www.genecards.org/), Online Mendelian Inheritance in Man (OMIM, https://www.omim.org/), and DisGeNET (https://www.disgenet.org/). Search terms included “acute myocardial infarction,” “myocardial infarction,” and “heart attack.” Retrieved genes were ranked by relevance scores, with high‐confidence targets selected based on composite scoring algorithms specific to each database.

#### 2.1.3. Protein–Protein Interaction (PPI) Network Construction

Common targets between EB active compounds and AMI‐related genes were identified through Venn diagram analysis, representing potential therapeutic intervention points. PPI networks were constructed using the STRING database (https://string-db.org/) Version 11.5 with high confidence interaction score threshold (> 0.7). Network visualization and topological parameter calculations were performed using Cytoscape software Version 3.9.1. Hub genes were identified based on composite ranking of degree centrality, betweenness centrality, and closeness centrality metrics.

#### 2.1.4. Functional Enrichment Analysis

Gene Ontology (GO) enrichment analysis encompassing biological processes (BPs), cellular components (CCs), and molecular functions (MFs) was conducted using the clusterProfiler *R* package. Kyoto Encyclopedia of Genes and Genomes (KEGG) pathway enrichment analysis identified significantly enriched signaling cascades associated with EB therapeutic mechanisms. Statistical significance was determined using hypergeometric distribution tests with Benjamini–Hochberg false discovery rate (FDR) correction, employing threshold criteria of *p* < 0.05 and *q* < 0.05.

### 2.2. scRNA‐seq Data Processing

#### 2.2.1. Data Acquisition and Quality Control

Single‐cell transcriptomic datasets of murine MI models were obtained from Gene Expression Omnibus (GEO) database under accession number GSE163465. It should be noted that this scRNA‐seq dataset was derived from untreated MI and normal control samples (*n* = 3 biological replicates per group, comprising 28,483 cells total) without EB intervention. Therefore, the single‐cell analysis serves to map the expression patterns and cellular distribution of predicted EB targets within the post‐infarction microenvironment, rather than directly demonstrating drug‐induced molecular alterations. Raw sequencing data underwent rigorous quality control using Seurat package (Version 4.3.0) in R environment. Cells with < 500 or > 5000 detected genes, < 1000 unique molecular identifiers (UMIs), or > 15% mitochondrial gene content were systematically excluded to eliminate low‐quality cells and potential doublets.

#### 2.2.2. Normalization and Dimensionality Reduction

Gene expression matrices were normalized using log‐normalization methodology implemented in Seurat. Highly variable features (top 2000 genes) were identified through variance‐stabilizing transformation to capture biologically relevant heterogeneity while reducing computational complexity. Data scaling was performed using ScaleData function to regress out technical confounders including mitochondrial percentage and cell cycle effects. Principal component analysis (PCA) was employed for linear dimensionality reduction, with optimal principal component number determined through elbow plot and jackstraw procedures. Uniform Manifold Approximation and Projection (UMAP) provided nonlinear dimensionality reduction for visualization of cellular relationships.

#### 2.2.3. Cell Clustering and Annotation

Unsupervised graph‐based clustering was performed using Louvain algorithm with resolution parameter optimized to 0.5, balancing cluster granularity and biological interpretability. Cell type annotation was accomplished through integration of canonical marker gene expression with SingleR automated annotation package (Version 1.10.0). Manual curation ensured annotation accuracy through examination of established cell type–specific markers: cardiomyocytes (Tnnt2, Myh6), cardiac fibroblasts (Col1a1, Pdgfra), endothelial cells (Pecam1, Cdh5), macrophages (Cd68, Adgre1), and T cells (Cd3e, Cd3g).

#### 2.2.4. Differential Expression and Functional Analysis

Cell type–specific differential gene expression analysis comparing MI versus control conditions was conducted using Wilcoxon rank‐sum test implemented in Seurat’s FindMarkers function. Genes exhibiting |log2(fold change)| > 0.5 and adjusted *p* value < 0.05 were classified as differentially expressed. GO and KEGG enrichment analyses of cell type–specific differentially expressed genes were performed using clusterProfiler, elucidating BPs and signaling pathways altered in post‐infarction myocardium.

#### 2.2.5. Pseudotime Trajectory Analysis

Cellular developmental trajectories and dynamic gene expression patterns were reconstructed using Monocle 3 package (Version 1.3.1). Dimensionality reduction was performed using UMAP, followed by trajectory graph learning through principal graph algorithms. Pseudotime ordering of cells along developmental paths enabled characterization of temporal gene expression dynamics during myocardial repair processes. Genes exhibiting significant expression changes along pseudotime (Moran’s I test, *q* < 0.05) were identified as temporally regulated transcripts.

#### 2.2.6. Integration of Network Pharmacology and Single‐Cell Data

Network pharmacology–predicted EB target genes were mapped onto single‐cell transcriptomic data to evaluate cell type–specific expression patterns. Feature plots and violin plots visualized expression distributions of predicted targets across cardiac cell populations. Correlation analysis between predicted targets and differentially expressed genes assessed the expression patterns of network pharmacology predictions and identified cell types that express predicted targets and are thus potentially responsive to EB intervention.

### 2.3. Statistical Analysis

All statistical analyses were performed using R software Version 4.2.0. Continuous variables were expressed as mean ± standard deviation. Comparisons between groups employed Student’s *t*‐test or Wilcoxon rank‐sum test depending on data distribution. Multiple testing correction utilized the Benjamini–Hochberg FDR method. Statistical significance threshold was established at *p* < 0.05 unless otherwise specified.

## 3. Results

### 3.1. Single‐Cell Transcriptomic Quality Assessment and Unsupervised Clustering Analysis

Employing 10× Genomics scRNA‐seq technology, we performed comprehensive transcriptomic profiling of murine cardiac tissues under MI and normal control conditions. Quality control metrics demonstrated comparable distribution patterns for both RNA feature detection (nFeature_RNA) and UMI counts (nCount_RNA) across experimental groups, validating robust data quality and inter‐sample comparability (Figures 1(a), 1(b), and 1(c)). Implementation of Seurat‐based unsupervised clustering algorithms successfully resolved multiple distinct cellular subpopulations, which exhibited clear spatial segregation patterns upon UMAP dimensionality reduction visualization (Figure 1(d)). Further comparative analysis revealed substantial alterations in cellular composition between post‐infarction and healthy myocardial tissues (Figures 1(e) and 1(f)), establishing the foundational cellular landscape for investigating EB therapeutic mechanisms. These findings provide critical cellular context for understanding how EB‐derived bioactive compounds may modulate inflammatory and reparative processes through multicellular coordination during AMI progression.

FIGURE 1Single‐cell transcriptomic data quality control and clustering analysis. (a–c) Quality control metrics for myocardial infarction (MI) and normal control samples, including RNA feature numbers, RNA counts, and sample quality assessment. (d) UMAP plot of cells based on Seurat clustering, with different colors representing distinct cell clusters. (e) UMAP plot grouped by cell types, showing spatial distribution of various cell subpopulations. (f) UMAP plot grouped by sample conditions, with blue representing MI samples and red representing normal samples.

**Figure:**
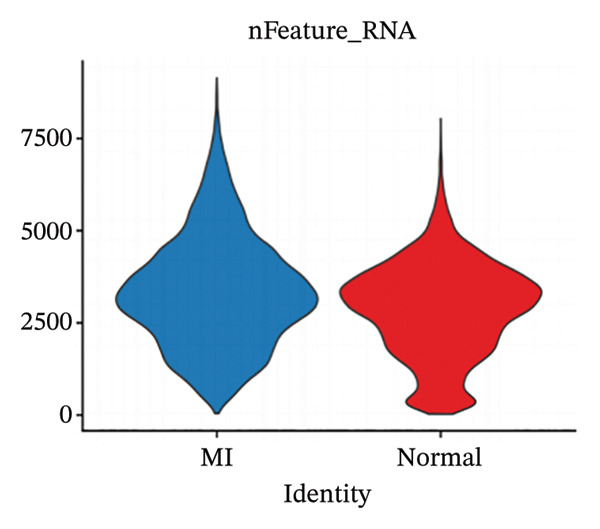
(a)

**Figure:**
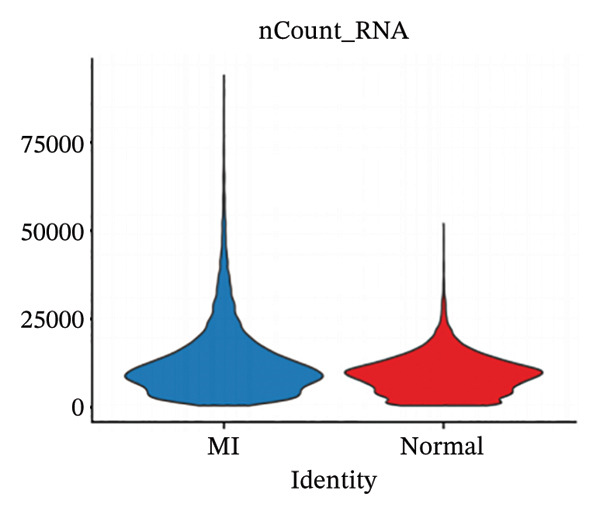
(b)

**Figure:**
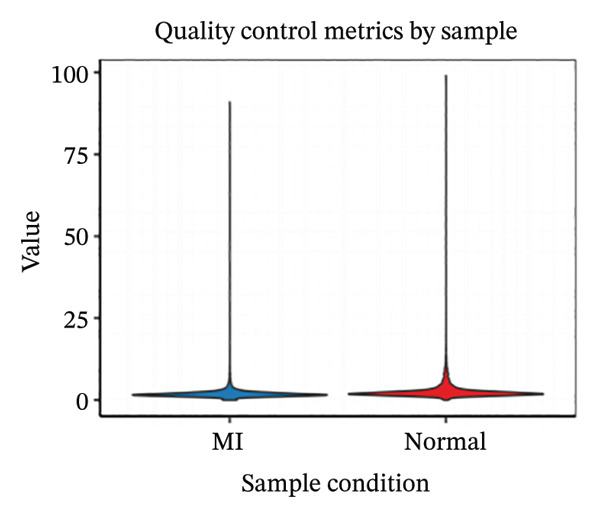
(c)

**Figure:**
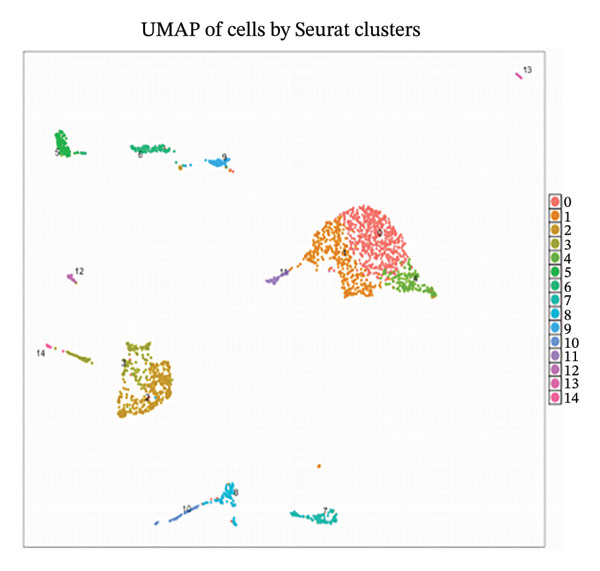
(d)

**Figure:**
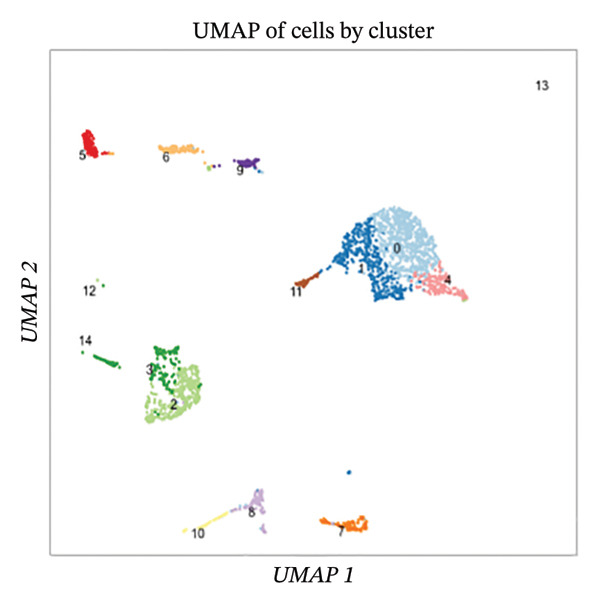
(e)

**Figure:**
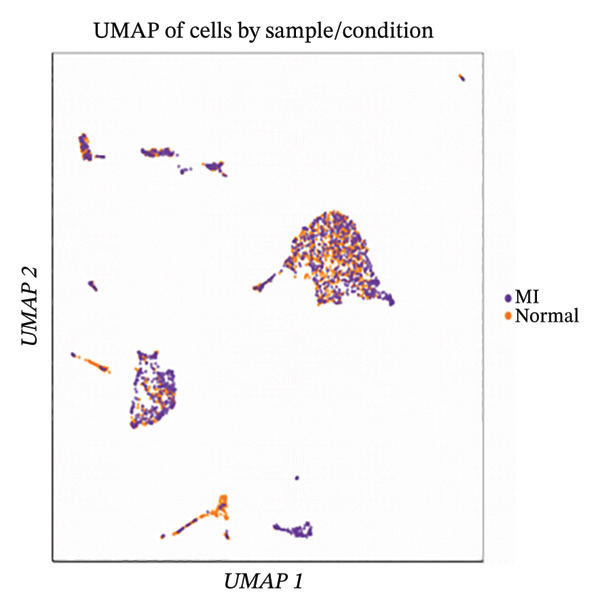
(f)

### 3.2. Cell Type Annotation and Compositional Dynamics in Post‐Infarction Myocardium

Integration of canonical marker gene expression profiles with automated annotation algorithms enabled comprehensive identification and characterization of diverse cardiac cell populations in both MI and control specimens. UMAP dimensionality reduction revealed distinct spatial organization of cells based on experimental conditions and cluster identities, demonstrating the robustness of our computational classification approach (Figures 2(a) and 2(b)). Systematic cell type annotation identified multiple cardiac cellular constituents including cardiomyocytes, cardiac fibroblasts, vascular endothelial cells, tissue‐resident and infiltrating immune cell populations, and additional stromal components (Figure 2(d)). Quantitative compositional analysis uncovered dramatic shifts in cellular proportions between pathological and physiological states, with specific cell types exhibiting pronounced abundance alterations (Figures 2(c) and 2(e)). Statistical assessment of cell type distributions across experimental conditions illuminated the dynamic cellular remodeling cascade characteristic of post‐infarction tissue reorganization (Figure 2(f)). These observations establish the heterogeneous cellular microenvironment wherein EB bioactive compounds may exert cell type–specific therapeutic effects, particularly through modulating inflammation‐associated cell populations and matrix‐producing fibroblasts during myocardial repair processes.

FIGURE 2Cell type identification and compositional analysis in cardiac tissues. (a) UMAP visualization of cells colored by sample condition, distinguishing between myocardial infarction (MI) and normal control samples. (b) UMAP plot showing cell clustering with distinct clusters represented by different colors. (c) Stacked bar chart displaying cell type composition across different conditions, illustrating the relative proportions of various cell populations. (d) UMAP plot with cells annotated by identified cell types, showing the spatial distribution of different cardiac cell populations. (e) Quantitative analysis of cell type proportions across conditions, with red and blue bars representing different sample groups. (f) Statistical analysis showing comparative metrics between experimental conditions (*n* = 3 biological replicates per group refers to 3 MI samples and 3 normal control samples from the original GSE163465 dataset, representing a total of 28,483 cells analyzed; note that this dataset does not include EB treatment groups).

**Figure:**
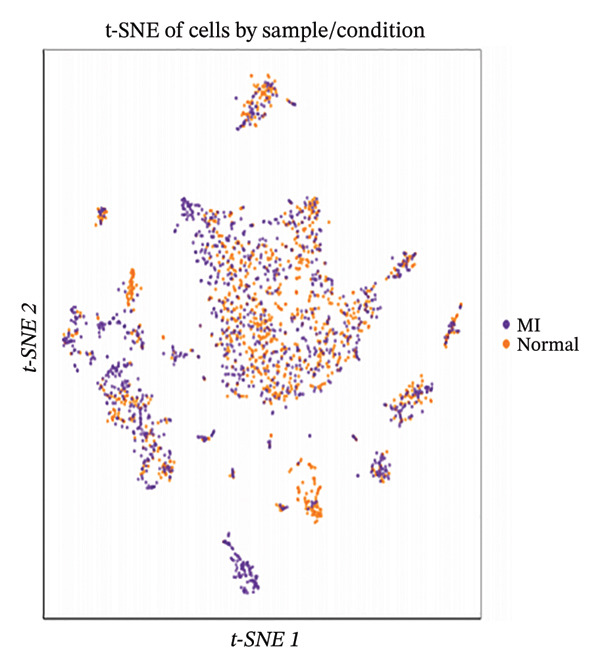
(a)

**Figure:**
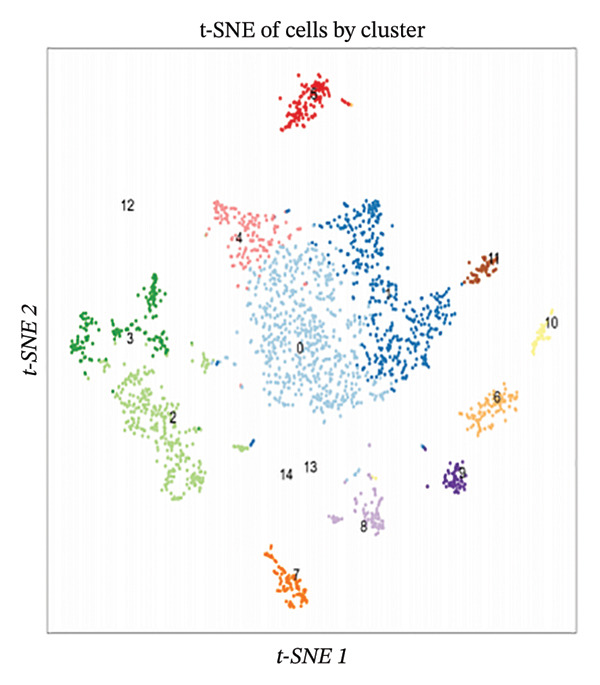
(b)

**Figure:**
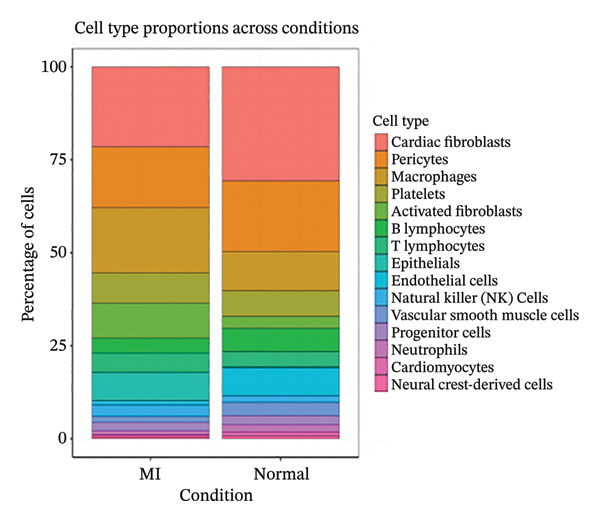
(c)

**Figure:**
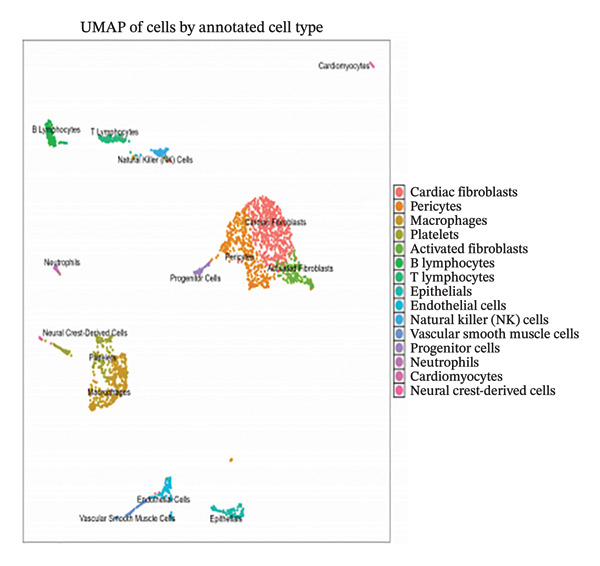
(d)

**Figure:**
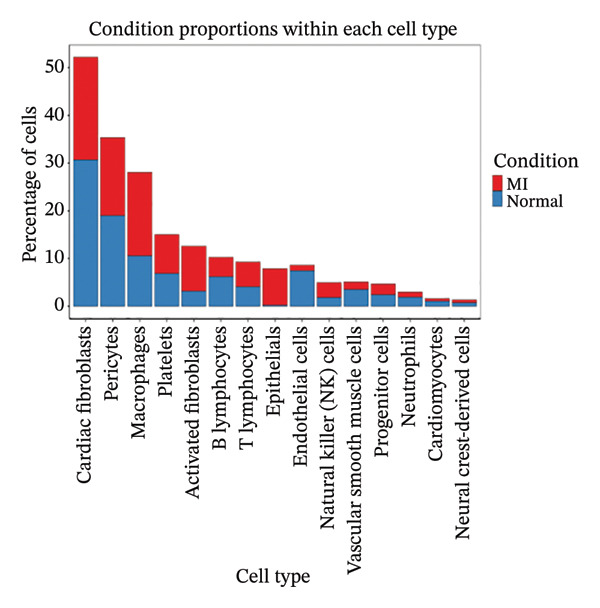
(e)

**Figure:**
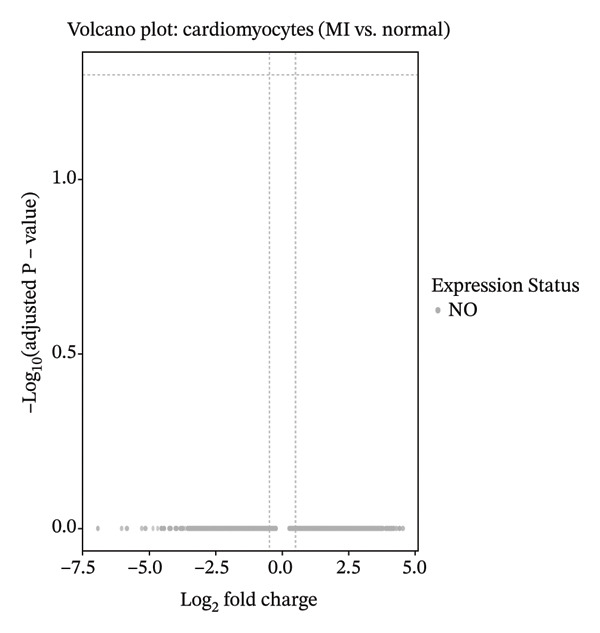
(f)

### 3.3. Transcriptional Reprogramming Signatures in Macrophage Populations During Myocardial Injury

Single‐cell resolution differential expression analysis unveiled distinctive transcriptional signatures characterizing macrophage populations isolated from infarcted myocardial tissues relative to healthy controls. Feature plot spatial visualization revealed systematic patterns of gene expression alterations, with significantly suppressed transcripts displaying diminished expression intensity (blue color gradients) and activated genes exhibiting enhanced expression levels (red color gradients) within discrete macrophage subpopulations. Analysis of downregulated transcripts identified suppression of homeostatic regulatory genes including Myh6 and Ptrf, which participate in maintaining normal cardiac contractile function and cellular metabolic homeostasis (Figure 3(a)). Conversely, examination of upregulated gene networks revealed enhanced expression of transcripts such as Gm19675 and Fhad1, potentially implicated in orchestrating inflammatory cascades and coordinating tissue remodeling responses during acute cardiac injury (Figure 3(b)). These comprehensive differential expression patterns demonstrate that macrophage populations undergo substantial transcriptional reprogramming following MI, potentially establishing cellular phenotypes susceptible to therapeutic modulation by EB compounds. The pronounced inflammatory polarization observed in post‐MI macrophages represents a critical cellular target through which EB’s anti‐inflammatory constituents may exert cardioprotective effects by attenuating excessive inflammation while preserving reparative macrophage functions essential for optimal tissue healing.

FIGURE 3Differential gene expression analysis in macrophages during myocardial infarction. (a) Feature plots showing the top downregulated genes in MI macrophages compared to normal controls. Each panel displays UMAP visualization with gene expression intensity represented by blue color gradients, where darker blue indicates higher expression levels. Representative genes include Myh6, Ptrf, and other significantly downregulated transcripts. (b) Feature plots demonstrating the top upregulated genes in MI macrophages. Expression levels are visualized using red color gradients, with darker red representing higher expression intensity. Notable upregulated genes include Gm19675, Fhad1, and other inflammation‐associated transcripts. Each plot represents the spatial distribution of gene expression across the macrophage population in the UMAP embedding.

**Figure:**
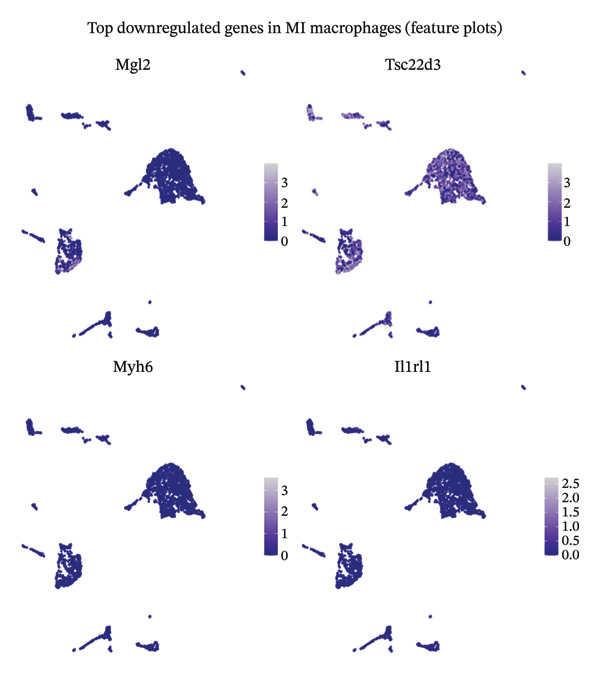
(a)

**Figure:**
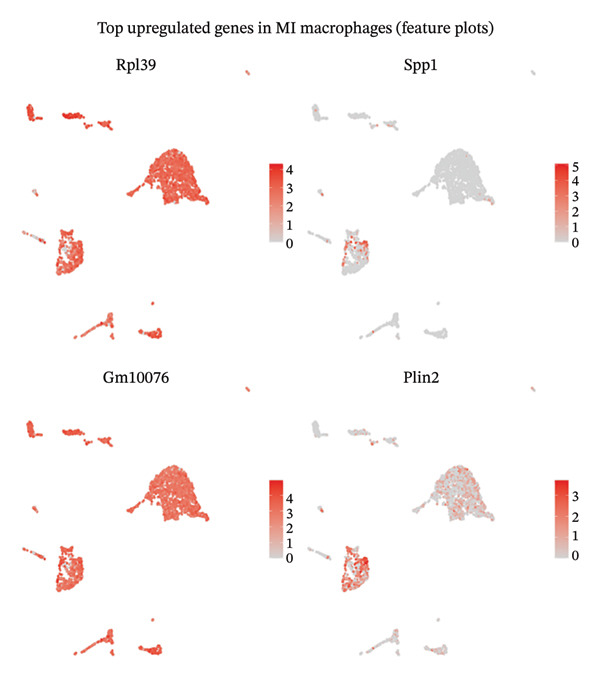
(b)

### 3.4. Comprehensive Differential Expression Profiling in Cardiac Fibroblasts and Immune Cell Populations

Systematic transcriptome‐wide differential expression analysis delineated distinct transcriptional landscapes characterizing both cardiac fibroblast and macrophage populations during MI compared to homeostatic conditions. Volcano plot representation of cardiac fibroblast gene expression revealed extensive transcriptional dysregulation, with numerous genes exhibiting statistically significant upregulation or downregulation relative to preestablished significance thresholds that distinguish biologically meaningful expression changes from technical variation (Figure 4(a)). Parallel analysis of macrophage populations demonstrated robust differential gene expression patterns, indicating comprehensive cellular reprogramming triggered by myocardial ischemic injury (Figure 4(b)). Feature plot spatial visualization of prominently upregulated genes in MI‐associated cardiac fibroblasts, including extracellular matrix components Sparc and Pyrc, revealed heterogeneous expression patterns within distinct fibroblast subpopulations (Figure 4(c)). These transcripts encode proteins critically involved in extracellular matrix reorganization, fibrotic tissue deposition, and inflammatory signal propagation, collectively suggesting phenotypic transformation of cardiac fibroblasts toward activated myofibroblast states during post‐infarction remodeling. The pronounced transcriptional alterations observed in these key cellular populations provide molecular evidence supporting the hypothesis that EB therapeutic mechanisms involve coordinated modulation of both inflammatory responses (via macrophage targeting) and fibrotic processes (through fibroblast regulation), thereby potentially limiting excessive scar formation while maintaining structural integrity necessary for preventing ventricular rupture.

FIGURE 4Differential gene expression analysis in cardiac cell populations during myocardial infarction. (a) Volcano plot showing differential gene expression in cardiac fibroblasts comparing MI versus normal conditions. The *x*‐axis represents log2 fold change, and the *y*‐axis shows ‐log10 (adjusted *p* value). Red dots indicate significantly upregulated genes, blue dots represent significantly downregulated genes, and gray dots show nonsignificant changes. (b) Volcano plot displaying differential gene expression in macrophages comparing MI versus normal conditions, with the same color coding scheme as (a). (c) Feature plots of the top upregulated genes in MI cardiac fibroblasts, including Sparc and Pyrc. Expression levels are visualized on UMAP projections with red color intensity indicating higher gene expression levels, while gray represents low or absent expression. Each panel shows the spatial distribution of gene expression across the fibroblast population.

**Figure:**
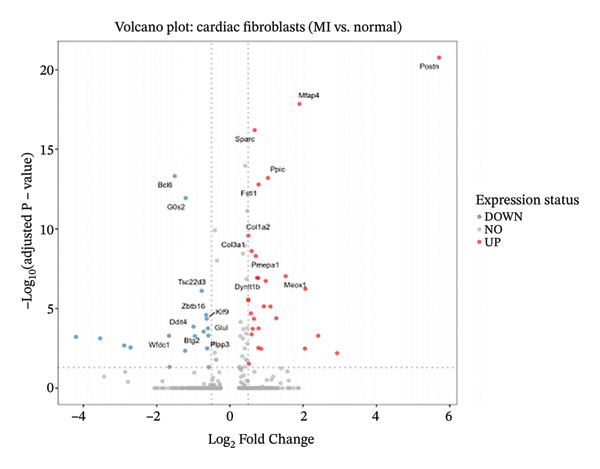
(a)

**Figure:**
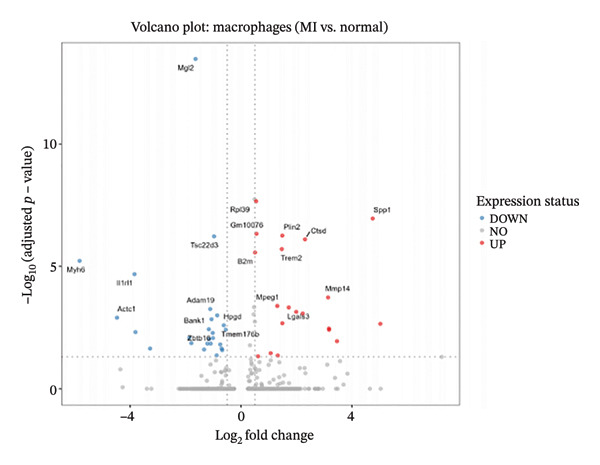
(b)

**Figure:**
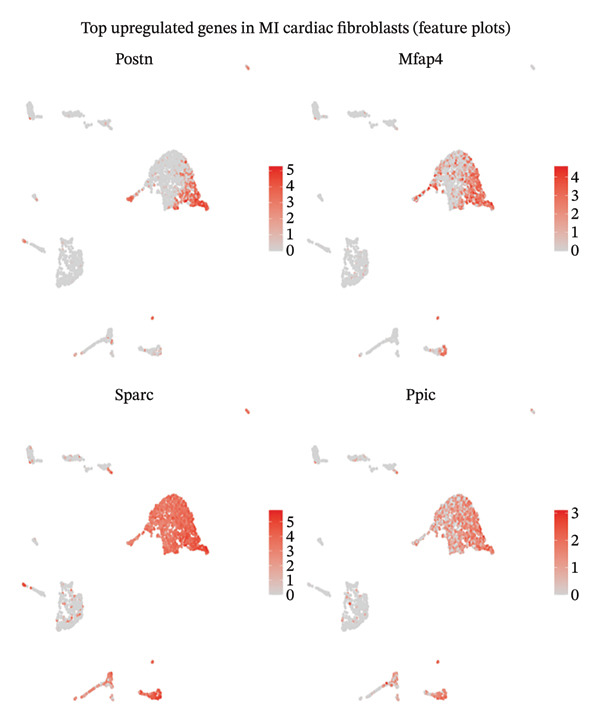
(c)

### 3.5. Functional Pathway Enrichment and Cell Type–Specific Transcriptional Dynamics

Comprehensive differential expression analysis within endothelial cell populations revealed substantial transcriptional remodeling during MI, as demonstrated through volcano plot visualization displaying characteristic distributions of activated and suppressed gene networks (Figure 5(a)). GO BP enrichment analysis performed on upregulated macrophage transcripts identified multiple critical functional categories, including programmed cell death regulation, proliferation suppression, and macromolecule metabolic process enhancement, collectively indicating activation of inflammatory and cellular stress response programs (Figure 5(b)). Enrichment of terms associated with angiogenic regulation and stress adaptation mechanisms demonstrates that macrophage populations execute multifaceted roles encompassing tissue repair coordination and remodeling orchestration during cardiac injury responses. Cell type–resolved feature plot analysis unveiled specific expression patterns, with endothelial populations exhibiting robust upregulation of stress‐adaptive genes including Nupr1 and Prx4, both implicated in cellular responses to oxidative damage and endoplasmic reticulum stress conditions (Figure 5(c)). Conversely, cardiac fibroblast populations demonstrated downregulation of developmental transcription factors Meox1 and Zeb1, suggesting alterations in differentiation programming and phenotypic plasticity potential (Figure 5(d)). These coordinated transcriptional changes spanning multiple cardiac cell lineages provide molecular evidence supporting the concept that EB exerts therapeutic effects through simultaneous modulation of diverse cellular processes across the cardiac microenvironment. Specifically, EB’s multicomponent composition may enable parallel targeting of endothelial stress responses, macrophage inflammatory activation, and fibroblast phenotypic transitions, collectively contributing to comprehensive cardioprotection during AMI.

FIGURE 5Functional enrichment analysis and cell type–specific differential gene expression. (a) Volcano plot showing differential gene expression in endothelial cells comparing MI versus normal conditions. The *x*‐axis represents log2 fold change, and *y*‐axis shows −log10 (adjusted *p* value). Red and blue dots indicate significantly up‐ and downregulated genes, respectively. (b) Gene ontology (GO) biological process enrichment analysis of upregulated genes in MI macrophages. Dot plot displays enriched pathways with dot size representing gene count and color intensity indicating statistical significance (GeneRatio on *x*‐axis). (c) Feature plots of top upregulated genes in MI endothelial cells, including Nupr1 and Prx4. Red color intensity indicates higher expression levels on UMAP projections. (d) Feature plots of top downregulated genes in MI cardiac fibroblasts, including Meox1 and Zeb1. Blue color intensity represents higher expression levels, with darker blue indicating stronger expression in the UMAP embedding.

**Figure:**
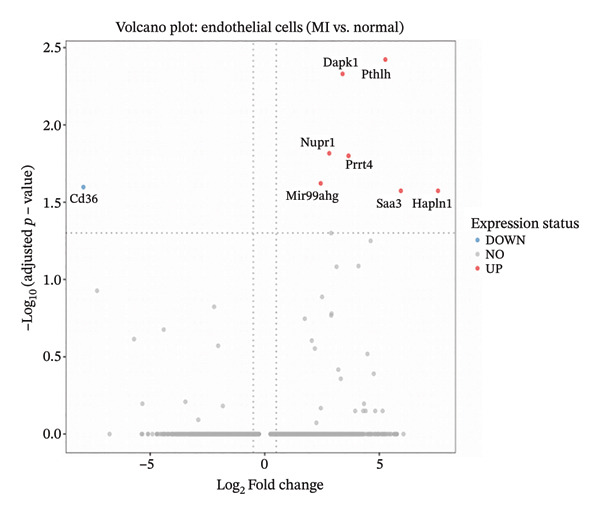
(a)

**Figure:**
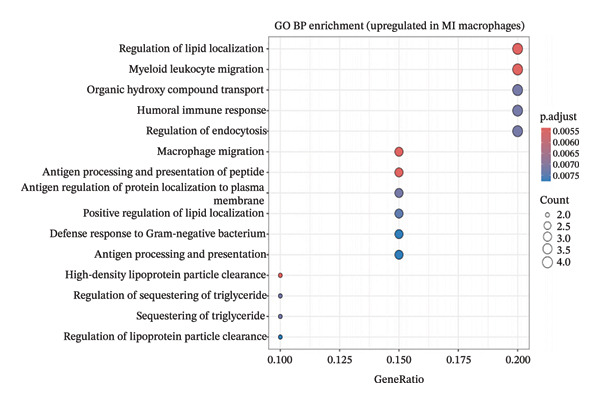
(b)

**Figure:**
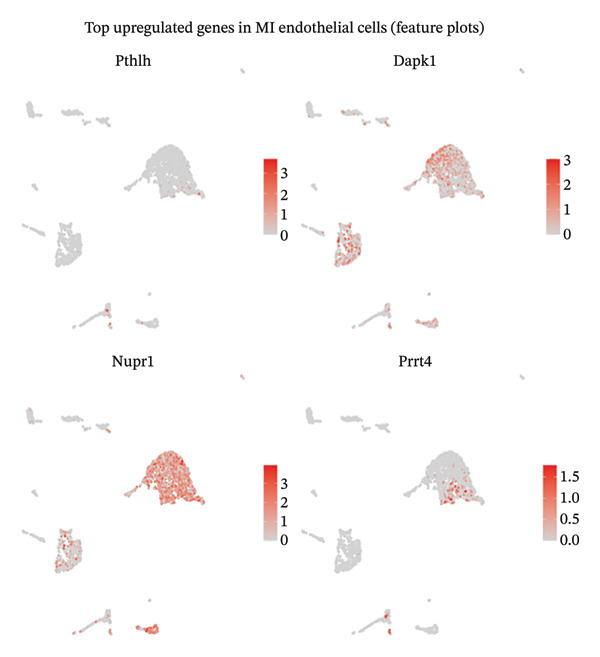
(c)

**Figure:**
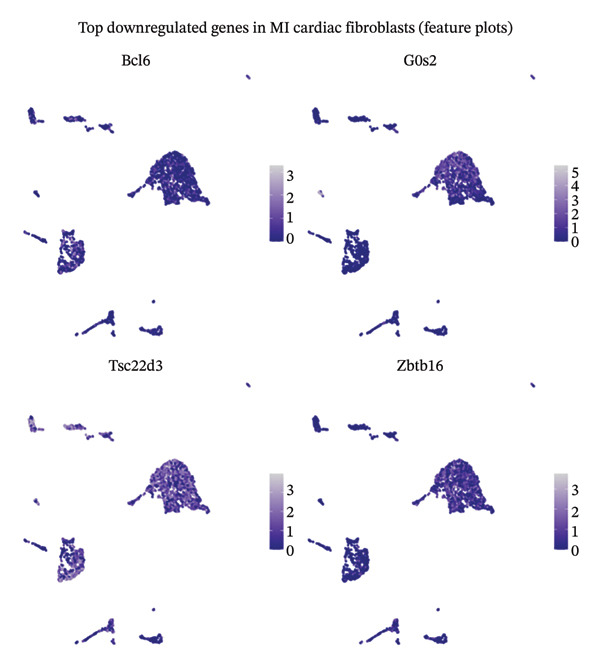
(d)

### 3.6. Cell Type–Resolved Functional Annotation Through Systematic GO Analysis

Systematic GO enrichment analysis performed across major cardiac cellular populations revealed cell type–specific functional signatures associated with MI pathogenesis and repair processes. Within cardiac fibroblast populations, transcriptionally activated genes demonstrated significant enrichment in BPs governing extracellular matrix assembly, collagen fibril organization, and wound healing cascades, indicating engagement of active tissue remodeling programs and initiation of fibrotic responses (Figure 6(a)). Cardiomyocyte populations exhibited enrichment in metabolic process categories, particularly those regulating energy production pathways and cellular stress adaptation mechanisms, reflecting the metabolic reprogramming necessitated by ischemic microenvironmental conditions (Figure 6(b)). Endothelial cell gene sets showed preferential enrichment in angiogenesis‐related BPs, vascular morphogenesis pathways, and endothelial cell motility programs, suggesting activation of compensatory revascularization attempts and vessel repair mechanisms (Figure 6(c)). Macrophage populations displayed prominent enrichment in inflammatory response cascades, including cytokine biosynthesis, immune cell recruitment, and phagocytic processes, underscoring their pivotal regulatory roles in the inflammatory cascade initiated by myocardial injury (Figure 6(d)). These comprehensive functional characterizations illuminate the cellular mechanisms through which EB bioactive constituents may exert multitargeted therapeutic effects. Specifically, EB compounds predicted to modulate inflammatory pathways (targeting macrophages), extracellular matrix remodeling (affecting fibroblasts), angiogenic processes (influencing endothelial cells), and metabolic adaptation (supporting cardiomyocytes) demonstrate convergence with the cell type–specific functional alterations identified through single‐cell transcriptomic profiling.

FIGURE 6Gene Ontology and KEGG pathway enrichment analysis in cardiac cell types. (a–d) GO biological process and KEGG pathway enrichment analysis for upregulated genes in different cardiac cell populations during myocardial infarction: (a) cardiac fibroblasts; (b) cardiac myocytes; (c) endothelial cells; (d) macrophages. Each dot represents an enriched pathway, with dot size indicating gene count and color intensity representing statistical significance (*p* value). GeneRatio (*x*‐axis) shows the proportion of genes in each pathway.

**Figure:**
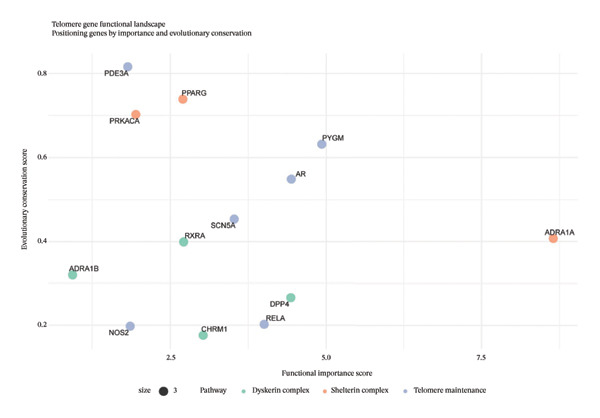
(a)

**Figure:**
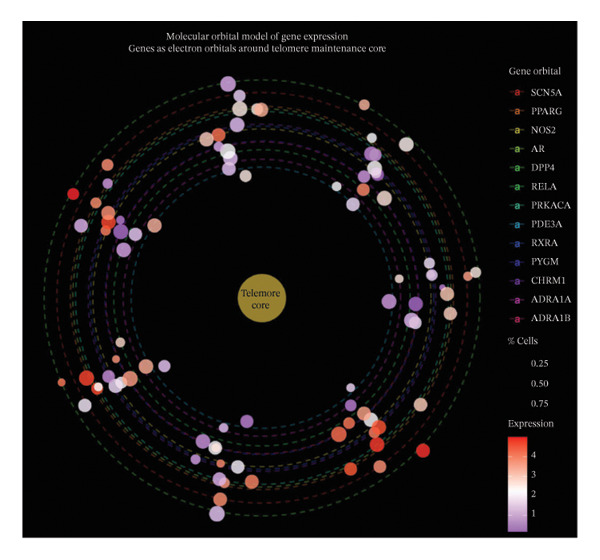
(b)

**Figure:**
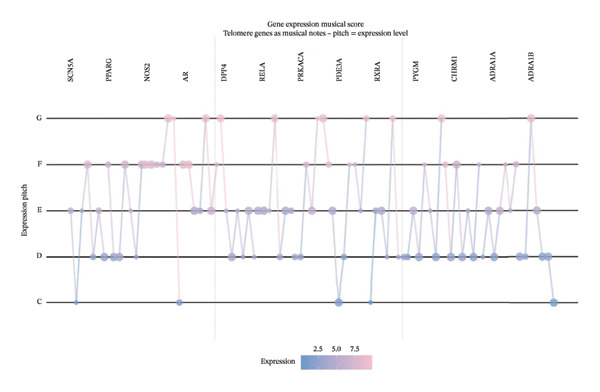
(c)

**Figure:**
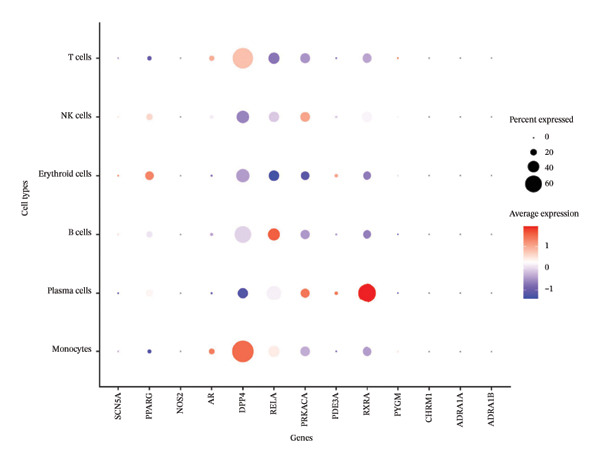
(d)

### 3.7. Pseudotemporal Trajectory Reconstruction Reveals Dynamic Cellular State Transitions

Monocle 3‐based pseudotime trajectory analysis successfully reconstructed dynamic cellular transition pathways characterizing MI progression and repair processes. Trajectory inference algorithms identified clear developmental trajectories featuring multiple decision nodes, representing critical cellular fate determination points occurring within the complex cardiac tissue microenvironment (Figures 7(a), 7(b), 7(c), and 7(d)). Cell type distribution analysis mapped along reconstructed pseudotime axes demonstrated ordered progression through discrete cellular states, with specific populations occupying characteristic temporal positions along the inferred developmental continuum (Figure 7(c)). Dynamic gene expression trend analysis identified key regulatory transcripts exhibiting systematic expression pattern changes across pseudotemporal progression, implicating their potential functional roles in governing cellular differentiation cascades and phenotypic state transitions (Figure 7(e)). Comparative trajectory analysis between experimental conditions revealed condition‐specific cellular developmental programs, highlighting how MI fundamentally alters physiological cardiac cell development trajectories and potentially promotes pathological cellular transitions (Figure 7(f)). These temporal analyses provide insights into the dynamic nature of cellular responses during myocardial repair, suggesting that EB therapeutic interventions may exert temporal stage‐specific effects by modulating cellular transition kinetics, potentially accelerating beneficial reparative transitions while suppressing pathological inflammatory or fibrotic progressions. The identification of discrete cellular states along reconstruction trajectories offers opportunities for precision timing of EB administration to maximize therapeutic efficacy during specific phases of post‐infarction remodeling.

FIGURE 7Pseudotime trajectory analysis of cardiac cell development. (a) Trajectory plots showing cellular developmental pathways under different conditions with pseudotime progression. (b) Overall cell population trajectory displaying the developmental continuum. (c) Cell type distribution along pseudotime trajectory with different colors representing distinct cell populations. (d) Alternative trajectory visualization showing cellular state transitions. (e) Gene expression trends along pseudotime showing dynamic expression patterns of key regulatory genes. (f) Condition‐specific trajectory analysis comparing normal versus MI cellular development patterns.

**Figure:**
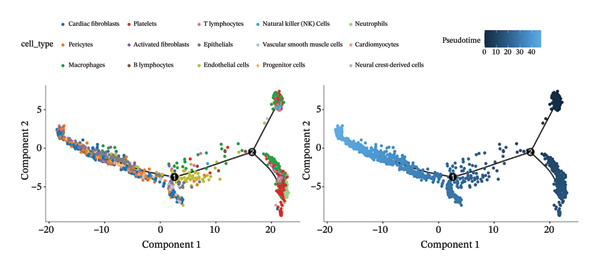
(a)

**Figure:**
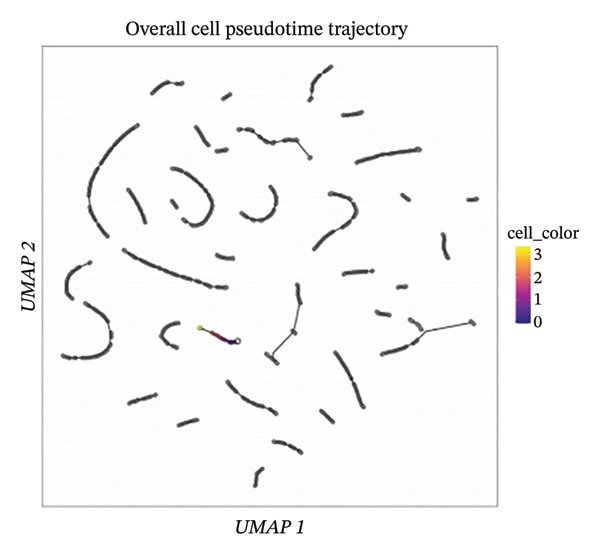
(b)

**Figure:**
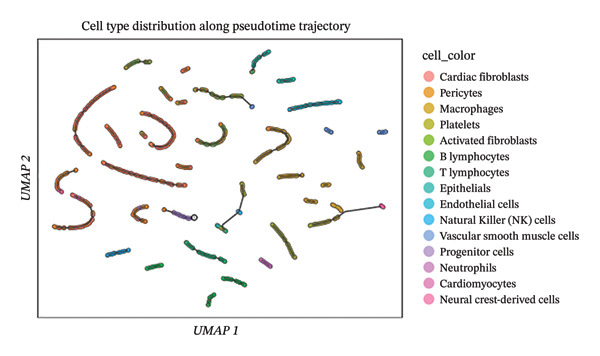
(c)

**Figure:**
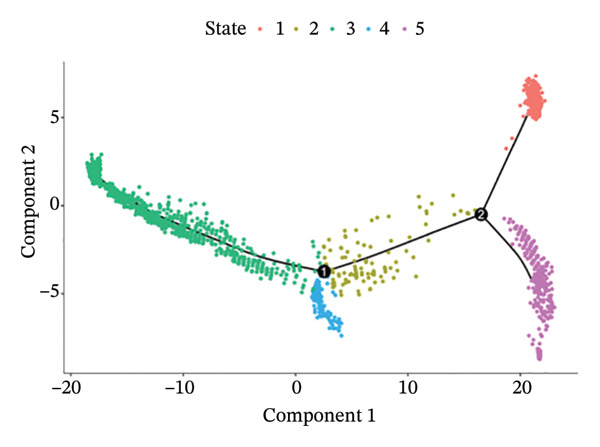
(d)

**Figure:**
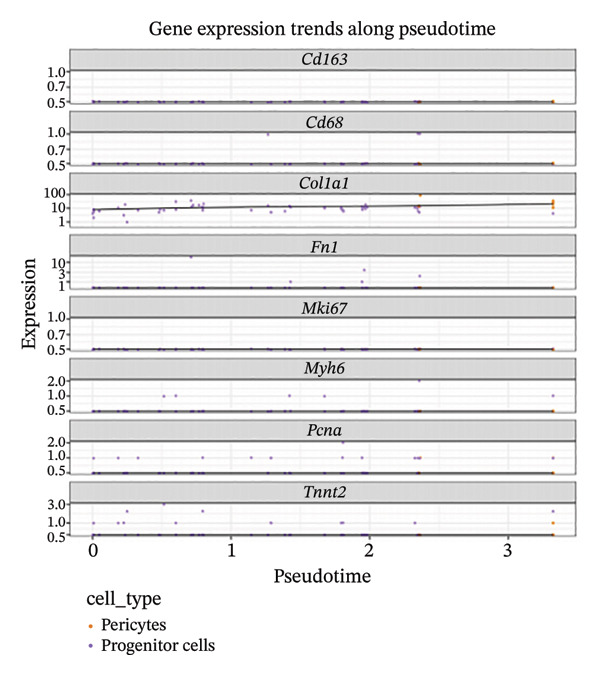
(e)

**Figure:**
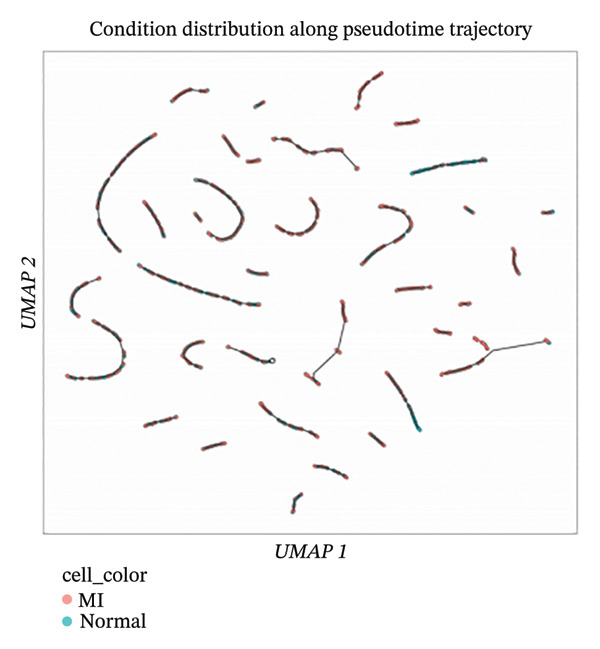
(f)

### 3.8. Complex Cellular Fate Mapping Through High‐Resolution Trajectory Analysis

Advanced pseudotemporal trajectory reconstruction revealed intricate cellular developmental architectures and complex state transition dynamics within cardiac tissues during MI pathogenesis. Pseudotime density distribution analysis demonstrated distinct temporal positioning for major cell types, with cardiac fibroblasts, macrophages, and endothelial cells occupying specific pseudotemporal niches along the reconstructed developmental continuum (Figure 8(a)). Multicondition comparative trajectory mapping unveiled divergent cellular developmental trajectories between physiological and pathological states, indicating disease‐induced perturbations in normal cellular fate determination processes (Figure 8(b)). Tree‐structured trajectory topologies revealed multiple bifurcation points and terminal differentiation states, reflecting the complex cellular differentiation cascades operating within the heterogeneous cardiac microenvironment (Figure 8(c)). Systematic gene expression dynamics analysis across trajectory‐defined cellular states identified coordinated transcriptional programs potentially governing cellular transitions and phenotypic switches relevant to cardiac repair and remodeling (Figure 8(d)). These comprehensive trajectory analyses provide critical insights into the temporal organization of cellular responses during MI, establishing a framework for understanding how EB compounds may influence cellular fate decisions. By identifying specific trajectory branch points where cellular phenotypes diverge between beneficial repair programs and detrimental pathological responses, these findings suggest potential therapeutic windows where EB intervention may optimally redirect cellular differentiation toward cardioprotective phenotypes. The integration of pseudotemporal dynamics with network pharmacology predictions enables hypothesis generation regarding which EB bioactive components may preferentially act at specific temporal stages of cellular reprogramming, facilitating development of optimized therapeutic strategies for AMI management.

FIGURE 8Cellular trajectory analysis and developmental state transitions. (a) Density plots showing pseudotime distribution of different cardiac cell types. (b) Trajectory analysis comparing cellular developmental pathways between normal and MI conditions with distinct cell populations marked by different colors. (c) Complex trajectory tree structure displaying cellular differentiation pathways and branching points. (d) Multipanel trajectory plots showing dynamic expression patterns of specific genes or markers across developmental states and conditions.

**Figure:**
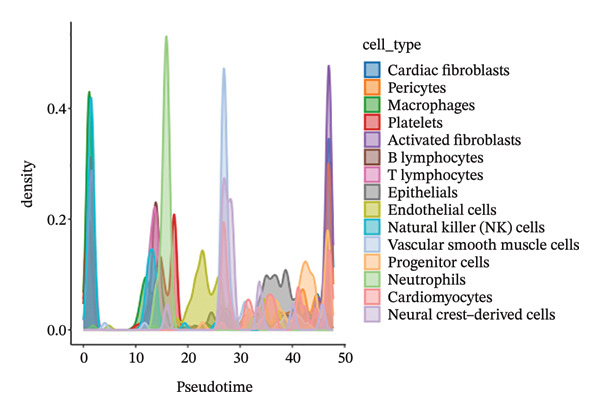
(a)

**Figure:**
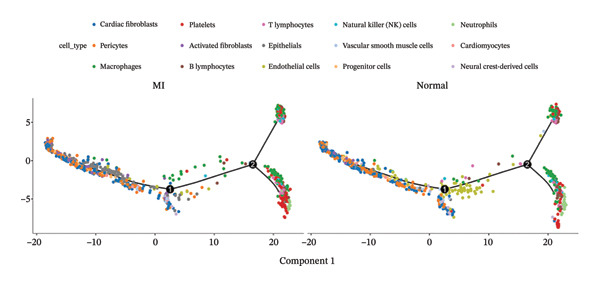
(b)

**Figure:**
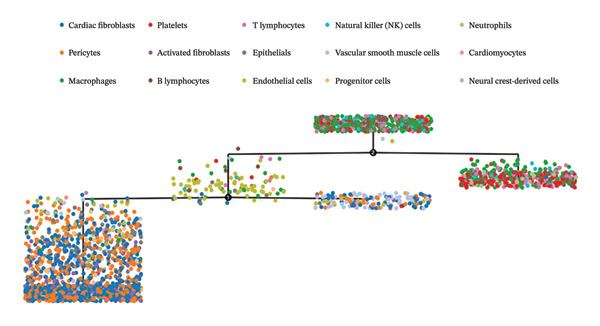
(c)

**Figure:**
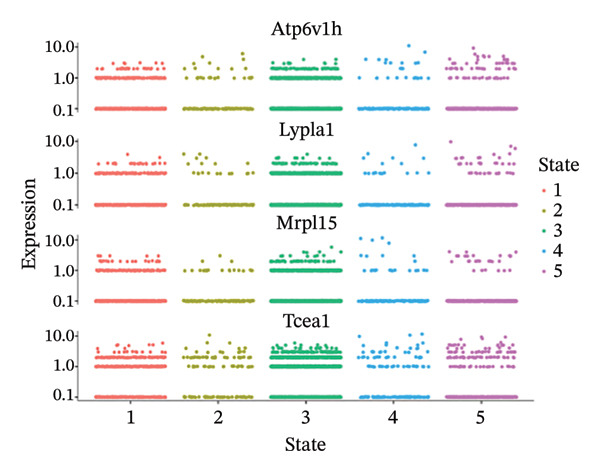
(d)

### 3.9. Cell–Cell Communication Network Analysis

To further investigate intercellular coordination within the cardiac microenvironment, we constructed global cell–cell communication networks under physiological and MI conditions.

In normal cardiac tissue, intercellular signaling exhibited a relatively balanced and organized communication architecture (Figures 9(a), 9(b), 9(c), 9(d), 9(e), 9(f), 9(g), and 9(h)). Multiple stromal, immune, endothelial, and parenchymal cell populations actively participated in ligand–receptor–mediated interactions, forming a coordinated signaling network that reflects homeostatic regulation of myocardial structure and function. Cardiac fibroblasts and endothelial cells displayed prominent outgoing signaling activity, suggesting their central roles in maintaining structural integrity and vascular homeostasis.

FIGURE 9Cell–cell communication network analysis in normal cardiac tissue. (a–h) Cell–cell communication networks in normal cardiac tissue showing outgoing signaling patterns from individual cell types. Each panel represents one specific sender cell population: (a) cardiac fibroblasts; (b) macrophages; (c) endothelial cells; (d) cardiomyocytes; (e) activated fibroblasts; (f) T lymphocytes; (g) B lymphocytes; (h) vascular smooth muscle cells.

**Figure:**
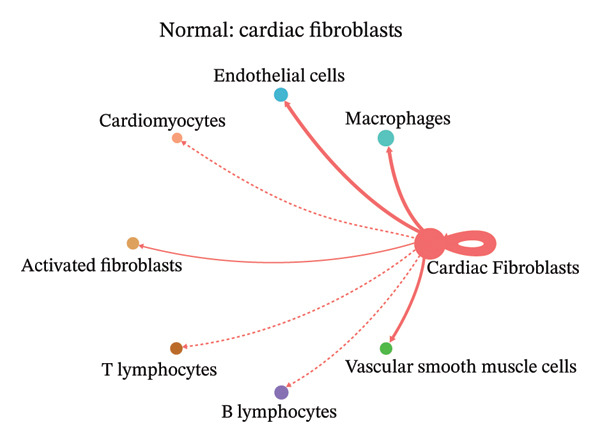
(a)

**Figure:**
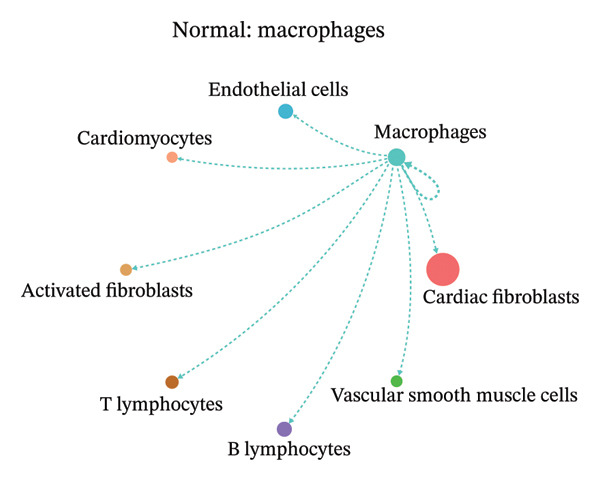
(b)

**Figure:**
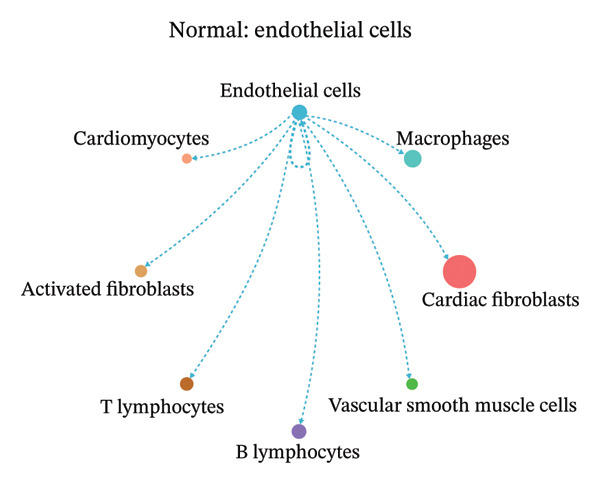
(c)

**Figure:**
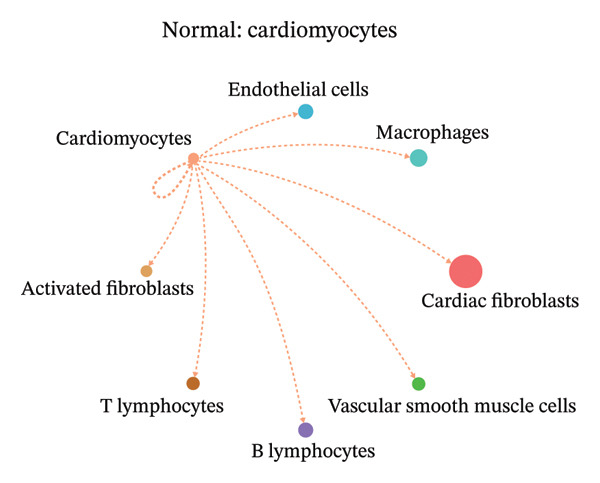
(d)

**Figure:**
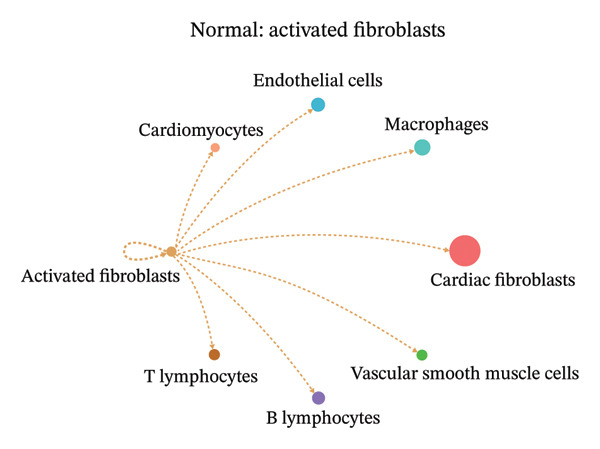
(e)

**Figure:**
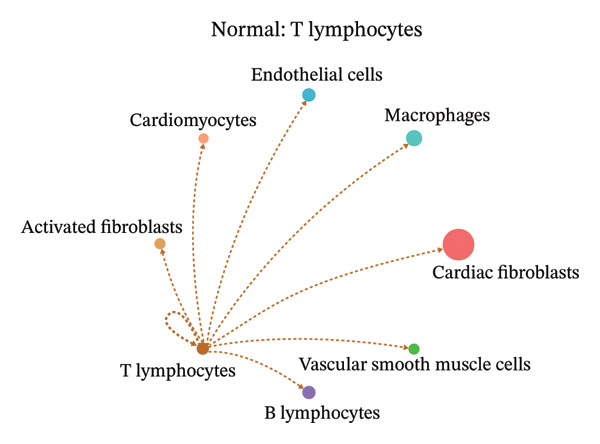
(f)

**Figure:**
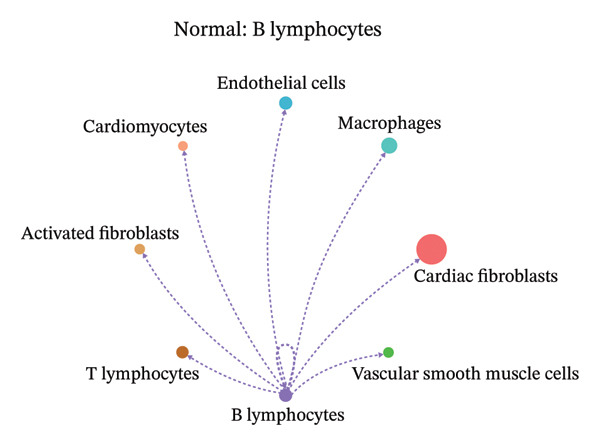
(g)

**Figure:**
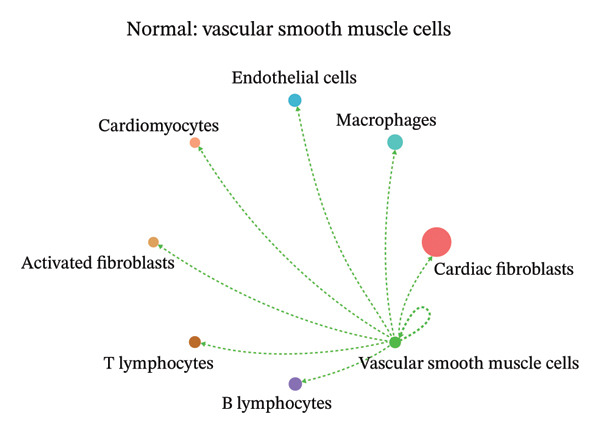
(h)

In contrast, MI samples demonstrated substantial remodeling of the communication network, characterized by altered interaction intensity and redistribution of signaling hubs. Notably, macrophages and activated fibroblasts exhibited markedly enhanced outgoing and incoming communication strength, indicating amplified inflammatory and matrix remodeling–related signaling (Figures 10(a), 10(b), 10(c), 10(d), 10(e), 10(f), 10(g), and 10(h)). The global network topology appeared more inflammation‐oriented, with strengthened interactions among immune and stromal compartments.

FIGURE 10Altered cell–cell communication networks in myocardial infarction. (a–h) Cell–cell communication networks in myocardial infarction (MI) tissue illustrating outgoing signaling from each cell type: (a) cardiac fibroblasts; (b) macrophages; (c) endothelial cells; (d) cardiomyocytes; (e) activated fibroblasts; (f) T lymphocytes; (g) B lymphocytes; (h) vascular smooth muscle cells.

**Figure:**
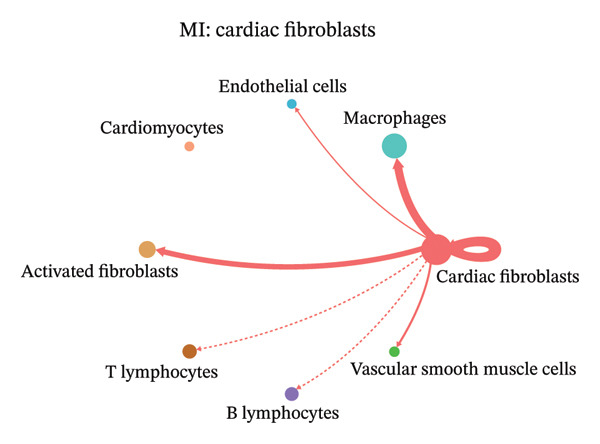
(a)

**Figure:**
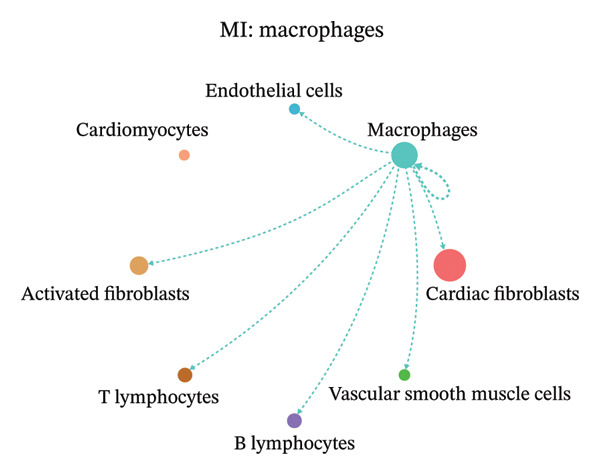
(b)

**Figure:**
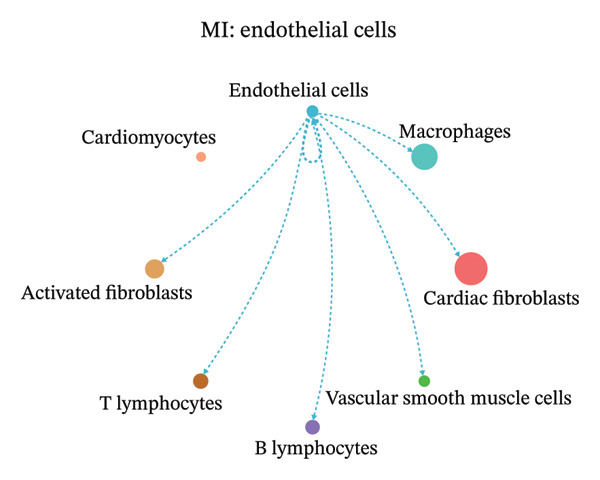
(c)

**Figure:**
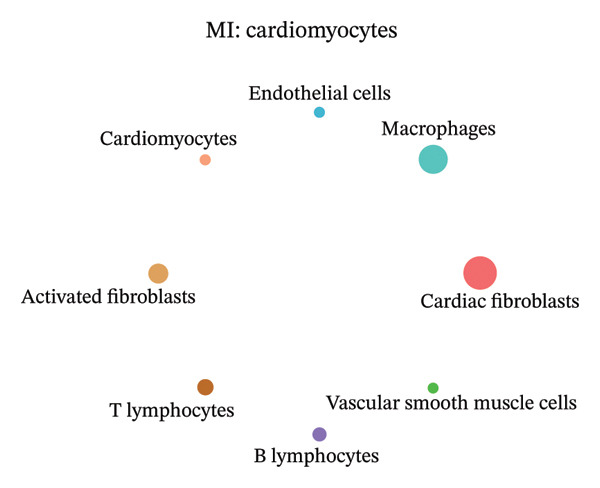
(d)

**Figure:**
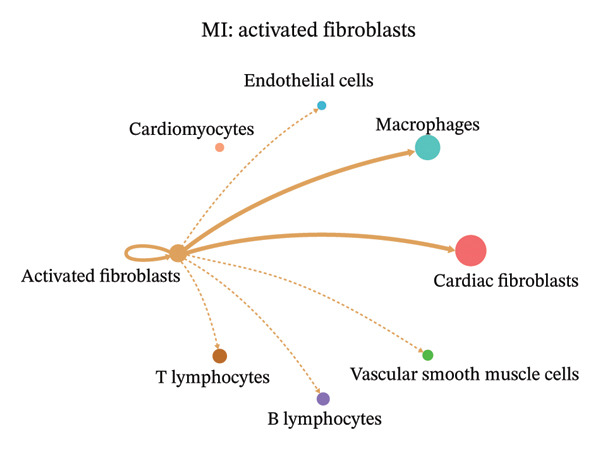
(e)

**Figure:**
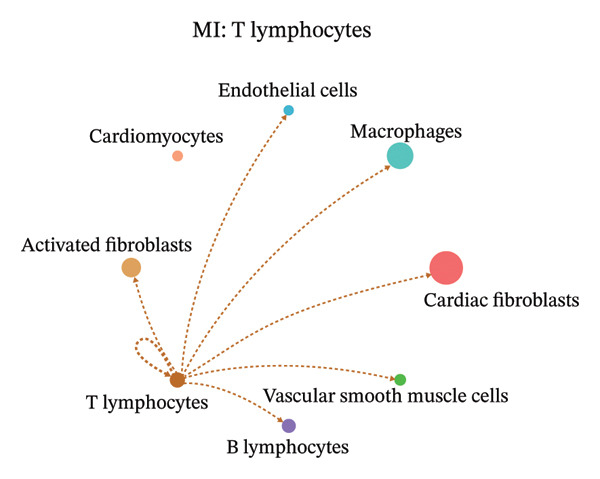
(f)

**Figure:**
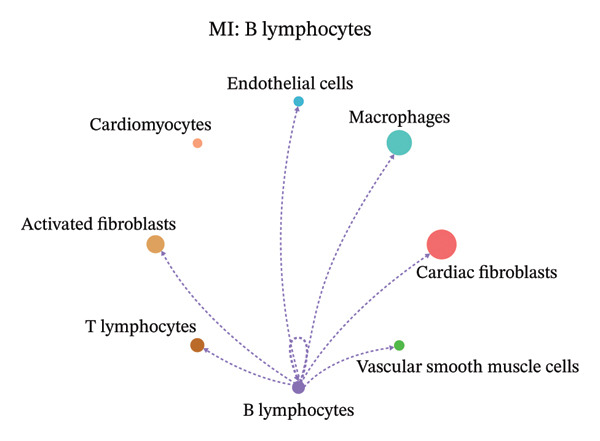
(g)

**Figure:**
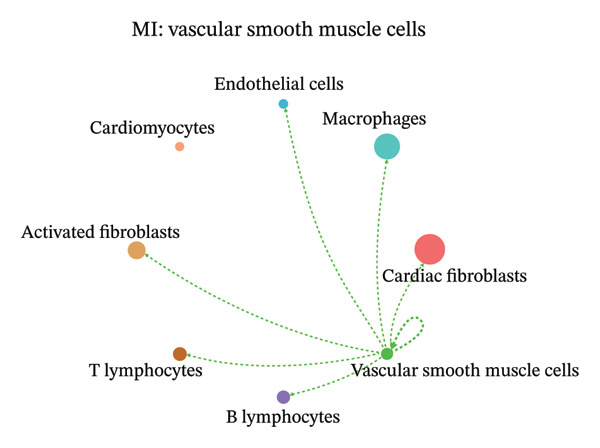
(h)

These findings suggest that MI induces a shift from a balanced homeostatic communication network toward an inflammation‐ and remodeling‐dominant intercellular signaling landscape, highlighting macrophages and activated fibroblasts as key regulators of post‐infarction microenvironmental reorganization.

## 4. Discussion

This investigation employed an integrated computational approach combining network pharmacology and single‐cell transcriptomics to systematically characterize the predicted molecular mechanisms underlying EB‐mediated cardioprotection in AMI. It should be emphasized that this study represents a hypothesis‐generating computational framework rather than definitive mechanistic elucidation. Our computational predictions suggest that EB may exert therapeutic effects through multicomponent, multitarget, multipathway mechanisms, potentially involving modulation of inflammatory responses, oxidative stress, angiogenesis, and extracellular matrix remodeling in diverse cardiac cell populations.

Network pharmacology analysis identified 45 predicted bioactive constituents from EB corresponding to 286 putative therapeutic targets, with 127 targets overlapping with AMI‐associated genes. PPI network topology revealed candidate hub genes including VEGFA, AKT1, TNF, IL6, and PTGS2, representing potentially critical nodes in EB’s pharmacological network. VEGFA functions as a master regulator of angiogenesis and vascular permeability, mediating therapeutic neovascularization in ischemic myocardium while potentially exacerbating vascular leak during acute inflammatory phases. AKT1 serves as a central mediator of PI3K‐AKT signaling, promoting cardiomyocyte survival, inhibiting apoptosis, and facilitating metabolic adaptation under stress conditions. TNF and IL6, as prototypical proinflammatory cytokines, orchestrate innate immune responses following myocardial injury, recruiting inflammatory cells while simultaneously activating reparative programs when properly regulated. PTGS2 (COX‐2) catalyzes prostaglandin synthesis, exerting context‐dependent effects on inflammation, pain, and tissue repair. The identification of these hub genes suggests that EB may modulate key regulatory nodes controlling inflammation–repair balance, which could potentially explain its therapeutic efficacy, though this requires experimental validation.

Single‐cell transcriptomic profiling provided unprecedented cellular resolution for characterizing post‐infarction myocardial remodeling. We successfully delineated heterogeneous cardiac cell populations and identified cell type–specific transcriptional alterations. Notably, macrophages and cardiac fibroblasts exhibited the most dramatic transcriptional reprogramming, consistent with their central roles in post‐infarction inflammation and repair. Macrophage analysis revealed upregulation of inflammatory mediators (cytokines and chemokines) and downregulation of homeostatic functions, indicating proinflammatory polarization. This finding aligns with established understanding that recruited monocyte‐derived macrophages initially adopt inflammatory phenotypes to clear necrotic debris, subsequently transitioning toward reparative phenotypes to facilitate tissue healing [10]. Cardiac fibroblasts demonstrated robust upregulation of extracellular matrix components (Sparc and collagens) and matrix remodeling enzymes, reflecting activation toward myofibroblast phenotypes driving scar formation [11]. While fibrotic responses provide structural support preventing ventricular rupture, excessive fibrosis contributes to adverse remodeling and heart failure progression [12].

Functional enrichment analyses illuminated BPs potentially targeted by EB intervention. KEGG pathway analysis highlighted PI3K‐AKT, MAPK, and TNF signaling cascades, which collectively govern cell survival, inflammation, and stress responses. PI3K‐AKT pathway activation promotes cardiomyocyte survival through inhibiting proapoptotic proteins and activating antiapoptotic factors [13]. MAPK cascades (ERK, JNK, and p38) transduce extracellular stimuli into transcriptional responses regulating proliferation, differentiation, and inflammation [14]. TNF signaling mediates both inflammatory injury and adaptive responses, with pathway outcomes depending on cellular context and signal duration [15]. The convergence of predicted EB targets on these pathways suggests potential coordinated modulation of multiple cellular processes underlying myocardial injury and repair, though experimental validation is required to confirm these predictions.

Pseudotime trajectory analysis revealed dynamic cellular transitions during myocardial repair, demonstrating progressive evolution from early inflammatory phases to later reparative phases. This temporal analysis identified stage‐specific gene expression programs governing cellular fate decisions, including inflammatory activation, matrix production, and tissue remodeling. The branching trajectory structures indicated heterogeneous cellular responses within cell populations, reflecting the complex and coordinated nature of tissue repair processes.

Integration of network pharmacology predictions with single‐cell validation data demonstrated that EB target genes exhibited preferential expression in inflammation‐associated cell types (macrophages and activated fibroblasts), supporting the hypothesis that EB modulates inflammatory and fibrotic responses. This cell type–specific targeting pattern may explain EB’s ability to balance inflammation control with preserved reparative capacity, a critical consideration for therapeutic interventions in AMI where excessive inflammation exacerbates injury while adequate inflammatory responses facilitate debris clearance and healing initiation.

Several limitations warrant acknowledgment. First, the primary limitation of this study is the absence of direct experimental validation. All 45 bioactive compounds and 127 predicted targets remain computationally derived without in vitro or in vivo confirmation. Key hub genes (VEGFA, AKT1, TNF, IL6, and PTGS2) require validation through western blotting, RT‐qPCR, or functional assays in EB‐treated MI models to substantiate the computational predictions. Second, a critical disconnect exists between network pharmacology predictions and single‐cell analyses. The GSE163465 dataset compares MI versus normal controls but contains no EB treatment groups. Therefore, our single‐cell analysis characterizes MI pathophysiology rather than directly demonstrating EB’s therapeutic effects. Mapping predicted targets onto scRNA‐seq data only shows expression patterns and potential cellular contexts for EB action, not actual drug‐induced transcriptional changes. Validation with EB‐treated single‐cell datasets represents essential future work. Third, methodological considerations include that our OB ≥ 30% and DL ≥ 0.18 threshold criteria, while based on established TCMSP conventions, are relatively lenient and may introduce false positives. The SwissTargetPrediction probability threshold of > 0.5 represents moderate stringency. Additionally, PPI network analysis relies solely on topological parameters without considering tissue‐specific relevance. Fourth, regarding multicomponent mechanisms, we acknowledge the challenge of distinguishing synergistic versus antagonistic effects in complex herbal formulations. Systems pharmacology approaches can identify potential interactions but cannot definitively prove synergy without experimental dose–response matrices. Fifth, pharmacokinetic considerations including oral bioavailability, plasma protein binding, tissue distribution, and metabolic transformation may substantially alter which predicted targets are actually engaged in vivo. For instance, while scutellarin demonstrates favorable OB and DL parameters computationally, its actual myocardial bioavailability and whether it reaches effective concentrations at predicted target sites remain uncertain without pharmacokinetic studies. Sixth, temporal dynamics considerations are important: our static scRNA‐seq snapshot cannot capture the temporal evolution of EB intervention effects across different MI phases (acute injury, inflammatory infiltration, proliferative repair, and maturation). Optimal intervention timing may differ for anti‐inflammatory versus proangiogenic effects. Seventh, translational challenges exist regarding species differences between the mouse‐derived scRNA‐seq data and human MI pathophysiology, including differences in immune cell composition, healing kinetics, and drug metabolism that may limit direct clinical translation. Most importantly, this study represents hypothesis‐generating computational research rather than definitive mechanistic elucidation, and all findings require extensive experimental validation including target confirmation, pathway functional studies, animal efficacy models, and ultimately clinical trials.

Despite these limitations, our integrated approach provides a comprehensive hypothesis‐generating framework into EB’s potential cardioprotective mechanisms, establishing a foundation for future investigations. Subsequent studies should focus on (1) experimental validation of predicted compound–target interactions through molecular docking, surface plasmon resonance, or cellular thermal shift assays; (2) functional characterization of identified pathways using genetic manipulation or pharmacological intervention in relevant disease models; (3) generation of EB‐treated single‐cell transcriptomic data to directly assess drug‐induced molecular alterations; (4) clinical translation through biomarker development and patient stratification strategies based on cellular composition or pathway activation signatures; and (5) optimization of EB formulations or active constituent combinations to maximize therapeutic efficacy while minimizing potential adverse effects.

## 5. Conclusion

Through systematic integration of network pharmacology and single‐cell transcriptomics, this investigation established a predictive computational framework characterizing the multicomponent, multitarget, multipathway mechanisms underlying EB‐mediated cardioprotection in AMI. Network pharmacology analysis identified candidate therapeutic targets and signaling pathways, while single‐cell transcriptomic analysis revealed cell type–specific expression patterns and dynamic cellular transitions. While these computational predictions provide valuable hypotheses for experimental investigation, definitive elucidation of EB’s cardioprotective mechanisms requires extensive validation including target confirmation, pathway functional studies, animal efficacy models, and ultimately clinical trials. Nevertheless, this framework offers rational guidance for prioritizing targets and cellular processes in future mechanistic research.

## Author Contributions

Lili Yan designed the study, performed network pharmacology analysis, conducted bioinformatics processing, and drafted the manuscript. Jun Cao contributed to single‐cell RNA‐seq data analysis, differential expression profiling, and functional enrichment analysis.

Qinglong Xie assisted in experimental design, data collection, and visualization and participated in manuscript revision. Lei Chen contributed to pathway analysis and validation of key targets and provided technical support in data interpretation. Yaqin Shi supervised the entire research process, provided critical revisions to the manuscript, and is the corresponding author responsible for the study.

## Funding

The authors have nothing to report.

## Ethics Statement

The authors have nothing to report.

## Conflicts of Interest

The authors declare no conflicts of interest.

## Data Availability

The data that support the findings of this study are available from the corresponding author upon reasonable request.
